# MeAJOR: Merged email assets from joint open-source repositories

**DOI:** 10.1016/j.dib.2026.112595

**Published:** 2026-02-16

**Authors:** Francisco Cardoso, João Vitorino, Paulo Mendes, Eva Maia, Isabel Praça

**Affiliations:** GECAD, ISEP, Polytechnic of Porto, Rua Dr. António Bernardino de Almeida, 4249-015 Porto, Portugal

**Keywords:** Phishing, Email, Machine learning, Deep learning, Cybersecurity, Dataset

## Abstract

Despite advancements, phishing remains a dominant force in the cybercrime field. Phishing emails continue to pose a significant threat to cybersecurity by exploiting human vulnerabilities to breach the systems defenses, resulting in financial losses, data theft and reputational damage to both victims and organizations being impersonated. While Machine Learning models are effective at detecting phishing threats, their performance mainly depends on the quality and diversity of the training data. This article presents MeAJOR (Merged email Assets from Joint Open-source Repositories) Corpus, a multi-source phishing email dataset designed to overcome critical limitations in existing resources. It integrates 108,685 samples representing a varied number of phishing tactics and legitimate emails, with several statistical features. Both the body and subject of the email entries are anonymized with standard tokens to ensure data privacy and confidentiality, while preserving the original context for email phishing detection models.

Specifications Table

**Table:** 

Subject	Computer Sciences
Specific subject area	Phishing email detection for Machine Learning and Deep Learning models
Type of data	Text, Table Filtered, Processed
Data collection	All data was collected from publicly available email spam and phishing datasets. The Nazario Phishing Corpus [1], Nigerian Fraud [2], TREC-05 [3], TREC-06 [4], and TREC-07 [5] datasets were combined, analysed, parsed, anonymized and new features were extracted, aiming to enhance the diversity of phishing examples and increase the overall sample size. These datasets were chosen because they provide both the original raw email messages, which allow greater flexibility in feature extraction, and the corresponding classification labels, making them suitable for phishing detection tasks.
Data source location	The data was extracted from diverse sources, some of which cannot be geolocated. The Nazario Phishing Corpus is from Nazario’s personal email inbox, who’s from Michigan, USA. The Nigerian Fraud dataset comprises of globally sourced fraudulent emails; hence no location could be found. The TREC-05, TREC-06, and TREC-07 datasets are from three different conferences (Text Retrieval Conference (TREC) Spam Evaluation Track) organised by NIST in Gaithersburg, Maryland, USA. The final dataset, its processing and storage were performed on the School of Engineering, Polytechnic of Porto, Portugal.
Data accessibility	Repository name: Zenodo Data identification number: 10.5281/zenodo.18471483 Direct URL to data: https://zenodo.org/records/18471483
Related research article	

## 1 Value of the Data

• This dataset provides a large collection of emails originating from both normal email activity and phishing campaigns, sourced from multiple existing public datasets with diverse attack scenarios.
• The complete information within each email has been anonymized to ensure data privacy and confidentiality, while also aiming to improve the generalization and transferability of email phishing detection solutions.
• The preprocessed features are obtained from email parsing, and different entities are replaced with standard tokens, to ease further analysis and feature engineering for model development.
• Researchers, artificial intelligence engineers, and cybersecurity professionals can benefit from this dataset to train, validate, and test their machine learning and deep learning solutions for email phishing detection systems.

## 2 Background

Phishing continues to be a dominant and destructive cybercrime. Recent data highlights the severity of this trend, with approximately 1.96 million attacks documented between May 2024 and April 2025 [6]. These attacks lead to significant financial losses worldwide while also compromising hundreds of thousands of accounts, primarily email [[7], [8], [9], [10]], with each breach costing an average of $4.4 million in 2025 [11]. Since email remains a primary channel for both personal and professional communication, it's a frequent target for these attacks. Given the constantly evolving threat, automated detection and mitigation strategies are important for protecting users and infrastructure. Machine learning (ML) approaches offer the potential for scalable and adaptive defenses against these evolving phishing campaigns. However, the effectiveness of these ML models is highly dependent on the quality and diversity of the data they are trained on.

While many open-source phishing repositories exist, recent research highlights significant limitations that prevent the development of robust machine learning models, including extreme class imbalance, restricted feature engineering, narrow coverage of phishing tactics, outdated samples, and inconsistent preprocessing methods [[12], [13], [14]]. Furthermore, many studies rely on private or small-scale datasets, which negatively impact the generalizability and reproducibility of their findings [15]. Because email repositories are saturated with Personally Identifiable Information (PII), legal and ethical hurdles often prevent the release of contemporary large-scale datasets. These challenges ultimately hinder the development of effective and realistic models, potentially leading to overfitting or inflated performance metrics when tested on small or biased datasets. Therefore, it is essential to provide a reliable and comprehensive dataset for email phishing detection.

## 3 Data Description

To ensure data privacy and confidentiality, all data is anonymized. The complete dataset is available in both a CSV file and a Parquet file, compressed with GZIP to improve usability. The features present in the *meajor_cleaned_preprocessed.csv* and *meajor_cleaned_preprocessed.parquet.gzip* files are described in Table 1.

**Table 1:** Feature descriptions.

Feature Name	Description
*source*	Original dataset from where the content came from (*trec5, trec6, trec7, nazario*, or *nigerian_fraud*)
*sender*	Hashed email address of the sender
*sender_domain*	Email domain of the sender
*receiver*	Hashed email address of the receiver
*receiver_domain*	Email domain of the receiver
*date*	Date and Time of when the email was sent (RFC 2822)
*subject*	Subject of the email, anonymized
*content_types*	Content type of the email body
*body*	Email body, anonymized
*urls*	URLs extracted from the email body
*url_count*	Number of URLs that were extracted
*url_length_max*	Maximum URL length
*url_length_avg*	Average URL length
*url_subdom_max*	Maximum URL subdomain count
*url_subdom_avg*	Average URL subdomain count
*attachment_count*	Number of attachments in the email
*has_attachments*	Binary flag indicating the presence of attachments (0 = no attachments; 1 = one or more attachments)
*attachment_types*	Types of attachments in the email
*language*	Language of the email (e.g. “en”)
*label*	Target binary label (0 = *benign*; 1 = *phishing*)

## 4 Experimental Design, Materials and Methods

The initial step involved collecting data from selected datasets and combining them into a single raw file. This raw dataset then underwent transformation to convert raw email data into a set of informative features suitable for Machine Learning models. Based on an analysis of commonly extracted features in recent research and their correlation with key components of phishing emails (such as body content, embedded links, attachments, headers, HTML structure, and domain reputation), a representative subset of features was selected from each category.

With the features defined and the unified dataset comprising 220,932 email samples, the subsequent and crucial step to ensure the quality and reliability of the analysis was data cleaning. This process is fundamental, especially when combining distinct phishing datasets from various sources, as the heterogeneous nature of emails can introduce noise that could negatively impact the development of robust models. Email messages come in diverse formats, character encodings, and content types. Therefore, to preserve the original content, the pipeline was designed to transform each raw email message into a consistent form while maintaining privacy safeguards.

The process begins by interpreting header fields (sender, receiver, date, and subject) through the decoding of various character encodings into legible text. This ensures that international characters (e.g., Chinese, Arabic, Greek, and Cyrillic) and special symbols are accurately rendered, preserving the nuances of the original message metadata.

Emails often consist of multiple parts, each with its own content type and body. These parts may be encoded using formats such as Base64 or quoted-printable, requiring individual handling. During preprocessing, any content transfer encoding is automatically detected and decoded. HTML markup is then stripped to isolate the underlying textual content, resulting in a clean and continuous stream of words suitable for further analysis.

Many email threads include quoted replies and forwarded content. The pipeline locates common reply separators and removes duplicate segments, isolating unique elements. By focusing on individual content, noise is reduced, and elements most relevant to phishing behavior are highlighted. Beyond text, emails often carry irrelevant decorations such as horizontal lines, separators, and special characters introduced by various clients. These artifacts are systematically removed. Additionally, look-alike characters, such as Unicode homoglyphs, are normalized to their standard ASCII counterparts. This normalization helps future text processing and removes elements that could be used to trick detection systems.

The preprocessing of the email body also includes the removal or masking of sensitive information. This encompasses email addresses, URLs, file paths, and phone numbers, which are replaced with standard placeholder tokens. This approach conceals actual user data while preserving the structural signals (e.g., the presence of a link) essential for phishing detection models. PGP signatures and emojis were also replaced solely for preserving structural signals. In addition to the text, metadata on attachments and hyperlinks is also gathered. Each attachment's type is recorded, and all links are extracted and analyzed for structural complexity. The replacement tokens are as follows:

• **[PGP]** – PGP signatures.
• **[EMOJI]** – Emojis.
• **[SYMBOL]** – Latin-1 supplement characters (Unicode range \U00A0-\U00BF).

In addition to masking sensitive information, an additional anonymization layer was implemented to further protect privacy within the open-source datasets, using a Small Language Model (SLM), specifically Gemma 3 27B. Each email message was preprocessed to replace real-world entities with descriptive tokens. This method allows for the obfuscation of specific identities while keeping the narrative context of the email as intact as possible. The entity anonymization tokens are as follows:

• **[NAME]** – Names (e.g., “John Smith”, “Jane”, “DeLay”).
• **[USERNAME], [INITIALS]** – Usernames & Initials (e.g., “jsmith77”, “J.S.”).
• **[EMAIL_ADDRESS]** – Email Addresses.
• **[PHONE_NUMBER]** – Phone & Fax Numbers.
• **[ADDRESS]** – Physical Addresses (streets, cities, zip codes).
• **[ORGANIZATION]** – Companies (e.g., “Enron”, “CNN”, “Berry Petroleum”).
• **[URL]** – URLs & Domain Names.
• **[IP_ADDRESS]** – IP Addresses.
• **[FILE_PATH], [FILE_NAME],[FILE]** – File Paths & Names.
• **[DATE], [TIME]** – Specific Dates & Times.
• **[FINANCIAL_INFO]** – Financial Data (e.g., “$250,000”, account numbers).
• **[PRODUCT]** – Branded Products/Services (e.g., “Apple”, “Windows”).
• **[REFERENCE_NUMBER]** – Unique IDs (e.g., ticket, serial numbers).

Finally, recognizing that email content may span multiple languages or include code snippets, the cleaned text is segmented, and language detection is applied to each part. These language labels can guide language-specific processing and enrich the feature set used by analytical models. After applying the processing pipeline to the combined dataset, some duplicate and empty samples were identified and removed. The resulting dataset is the preprocessed version of the MeAJOR Corpus, comprising a total of 108,685 emails.

From the resulting features, *url_count, url_length_max, url_length_avg, url_subdom_max, url_subdom_avg, attachment_count, has_attachments, attachment_types*, are ready to use. The subject and body features, both anonymized and not, are parsed and ready for further processing to be used on specific ML or DL models.

## Limitations

Despite this dataset having multiple statistical features, they are not very exhaustive, as the work was focused more on extracting and preprocessing the main characteristics of each email. Future work plans to improve on feature engineering, making the dataset more suitable for direct applications.

## Ethics Statement

The authors confirm that they have read and followed the ethical requirements for publication in Data in Brief. This work does not involve human subjects, animal experiments, or any data collected from social media platforms. This work involves data collected from publicly available cybersecurity repositories, which was anonymized to remove personally identifiable information.

## CRediT Author Statement

**Francisco Cardoso:** Anonymization, Data Curation, Validation, Writing. **João Vitorino:** Validation, Investigation, Writing. **Paulo Mendes:** Methodology, Data Curation, Writing. **Eva Maia:** Conceptualization, Investigation, Supervision. **Isabel Praça:** Conceptualization, Resources, Project administration, Funding acquisition.

## Data Availability

ZENODOMeAJOR: Merged email Assets from Joint Open-source Repositories (Original data)
